# Suitability of Text-Based Communications for the Delivery of Psychological Therapeutic Services to Rural and Remote Communities: Scoping Review

**DOI:** 10.2196/19478

**Published:** 2021-02-24

**Authors:** Anne Dwyer, Abílio de Almeida Neto, Dominique Estival, Weicong Li, Christa Lam-Cassettari, Mark Antoniou

**Affiliations:** 1 The MARCS Institute for Brain, Behaviour and Development Western Sydney University Penrith Australia; 2 Centre for Work Health and Safety NSW Government Gosford Australia

**Keywords:** mental health services, text messaging, counseling, mobile health, natural language processing

## Abstract

**Background:**

People living in rural and remote areas have poorer access to mental health services than those living in cities. They are also less likely to seek help because of self-stigma and entrenched stoic beliefs about help seeking as a sign of weakness. E-mental health services can span great distances to reach those in need and offer a degree of privacy and anonymity exceeding that of traditional face-to-face counseling and open up possibilities for identifying at-risk individuals for targeted intervention.

**Objective:**

This scoping review maps the research that has explored text-based e-mental health counseling services and studies that have used language use patterns to predict mental health status. In doing so, one of the aims was to determine whether text-based counseling services have the potential to circumvent the barriers faced by clients in rural and remote communities using technology and whether text-based communications, in particular, can be used to identify individuals at risk of psychological distress or self-harm.

**Methods:**

We conducted a comprehensive electronic literature search of PsycINFO, PubMed, ERIC, and Web of Science databases for articles published in English through November 2020.

**Results:**

Of the 9134 articles screened, 70 met the eligibility criteria and were included in the review. There is preliminary evidence to suggest that text-based, real-time communication with a qualified therapist is an effective form of e-mental health service delivery, particularly for individuals concerned with stigma and confidentiality. There is also converging evidence that text-based communications that have been analyzed using computational linguistic techniques can be used to accurately predict progress during treatment and identify individuals at risk of serious mental health conditions and suicide.

**Conclusions:**

This review reveals a clear need for intensified research into the extent to which text-based counseling (and predictive models using modern computational linguistics tools) may help deliver mental health treatments to underserved groups such as regional communities, identify at-risk individuals for targeted intervention, and predict progress during treatment. Such approaches have implications for policy development to improve intervention accessibility in at-risk and underserved populations.

## Introduction

### Defining E-Mental Health Services

E-mental health is an umbrella term used to describe services delivered via online, mobile, or phone-based platforms to diagnose, treat, or prevent mental health conditions, such as depression, anxiety, and substance abuse. This burgeoning field includes websites offering static information and resources such as Headspace and Beyond Blue [1,2], peer support services such as 7 Cups [3], interactive counseling services via telephone such as Kids Help Line [4], videoconferencing, email, and text-based chat (eg, SMS). With numerous approaches to e-mental health, some services use several modes of delivery (eg, Mental Health Online offers therapy via email, chat, and video). Technology has evolved rapidly, and the number of e-mental health services is growing, but without a rigorous evaluation of service delivery, much remains unknown.

### Context and Scope for the Review

E-mental health services can potentially fill an important service gap in the context of improving mental health outcomes for communities outside major cities. In Australia, these account for 28% of the population (over 7 million people) [5]. Living in rural and remote communities is associated with poorer health outcomes, including mental illness. As remoteness increases, the prevalence of mental illness and the incidence of self-harm and suicide also increase [6]. Suicide rates are 40% higher in rural areas than in cities, and double in remote areas, whereas suicide rates in regional and rural areas are increasing faster than in capital cities [7]*.* Farmers are at an increased risk of suicide compared with other occupations in rural and remote communities [8-10]. People living in rural areas generally have poorer access to health services than those living in cities for several reasons. First, fewer mental health professionals work in rural and remote areas [11], rendering access to mental health services more difficult owing to time requirements, distance, and limited service capacity. Public transport is often not viable in low-density rural areas, which is particularly problematic in Australia [12]. Second, stigma toward mental health issues becomes a help-seeking barrier in smaller communities, because of fears of compromised confidentiality and being the topic of community gossip [13]. This may be more pronounced for males, for whom self-stigma and entrenched stoic beliefs about help seeking as a sign of weakness can delay psychological counseling or prevent engagement with mental health services altogether [13,14]. Per capita needs for mental health services in rural and remote areas are at least as great as in cities; therefore, these barriers to mental health service delivery are a cause for concern. Through this lens of concern, we approach our review.

With the proliferation of smartphones, mental health services can use technology to circumvent barriers and span great distances to reach individuals. E-mental health initiatives can potentially make a valuable contribution to service delivery in rural areas, although more research is needed [15]. Two aspects of text-based counseling services are especially likely to appeal to people living in rural and remote communities. First, the potentially anonymous nature of text-based interactions with therapists will appeal to individuals who are concerned with stigma and are reluctant to engage with face-to-face services. Second, text-based counseling allows clients to interact in real time with a qualified therapist at a time convenient for them, without the need to take time off work. Encouragingly, there is evidence that clients who have accessed e-mental health services show comparable outcomes to those in traditional face-to-face counseling services, as described previously [16].

### Objectives

This scoping review maps the research that has explored text-based e-mental health counseling services and studies that have used language use patterns to predict mental health status. Our search considered publications in both peer-reviewed and gray literature to capture existing and emerging research within the field. In addition to providing an overview of the literature, this review aims to identify priorities for future research. As will become clear, text-based communications lend themselves to predictive analyses more readily and on a larger scale than spoken communication.

## Methods

### Overview

We performed a scoping review following the PRISMA (Preferred Reporting Items for Systematic Reviews and Meta-analyses) Extension for Scoping Reviews checklist (Multimedia Appendix 1). A scoping review protocol was created to guide the process and is available from the corresponding author upon request.

### Eligibility Criteria

It was decided that relatively broad inclusion criteria would be used to consider all publications with reference to the relevant mental health conditions (ie, depression, suicidal ideation, and anxiety), online counseling, and linguistic indicators for psychological conditions. All publication types were available for selection (journal articles, conference proceedings, reports, and unpublished theses). Therefore, studies were included if they (1) involved an intervention that occurred in real time, (2) were text-based, and (3) involved a qualified human clinician.

Publications were also considered for inclusion if they were related to mental health or counseling more generally if they had a text-based component. In addition, studies were included if they examined whether text patterns could be used to predict mental health status. Only articles written in English were included in this study.

Our scope is limited to the literature that examined e-mental health approaches involving text-based, real-time communication with a qualified human therapist and the predictive power of language use patterns. Within the eHealth sector, SMS text messaging and email are increasingly used for mental health care [17], medication adherence [18], treatment compliance [19], aftercare support [20], and reminders and supportive messages between face-to-face visits [21,22]. Encouragingly, virtual interventions incorporating scheduled messages or emails to support client participation have shown reductions in symptoms of depression, anxiety, and stress [21,23-25]. However, text-based technologies or the provision of mental health services by virtual agents or artificial intelligence algorithms cannot capture the dynamic back-and-forth communication exchanges of interest here and are not covered in the review. Therefore, studies that fell into the above categories were excluded.

### Information Sources

Articles were identified by searching through the following databases: PsycINFO, PubMed, ERIC, and Web of Science.

### Search Strategy

A literature search was conducted in July 2019 and updated in November 2020. The following search terms were used: (“therap*” OR “counsel*” OR “intervention” OR “depress*” OR “suicid*” OR “anxiety”) AND (“internet” OR “text” OR “linguistic” OR “word use” OR “natural language processing”). The search strategy was modified according to the different databases. The databases were searched from inception to November 24, 2020. As a final step, the reference lists contained within selected publications were searched for other relevant publications.

### Selection of Sources of Evidence

Screening is a two-step process. AD removed duplicates and screened titles and abstracts to determine relevance to the topics of mental health and text-based counseling. These publications were then read in full to determine their relevance to the research question. CLC and MA checked the excluded studies and confirmed that the exclusion criteria were applied correctly.

### Data Charting Process

We created a standardized template to extract relevant data from the included studies, such as publication type, journal name, reference to text-based e-mental health (or its variants) in the article title, reference to psychological conditions in the publication title, country of focus of the research, geographic location of data collection (urban, rural, etc), sample size of individual groups, and the gender composition of the sample. Data were extracted by AD, DE, and CLC and cross-checked by MA to ensure correctness and completeness.

### Data Analysis and Synthesis

We synthesized the collated data using descriptive statistics (frequencies and proportions). Microsoft Excel was used to analyze the data.

## Results

### Study Selection

**Figure 1:**
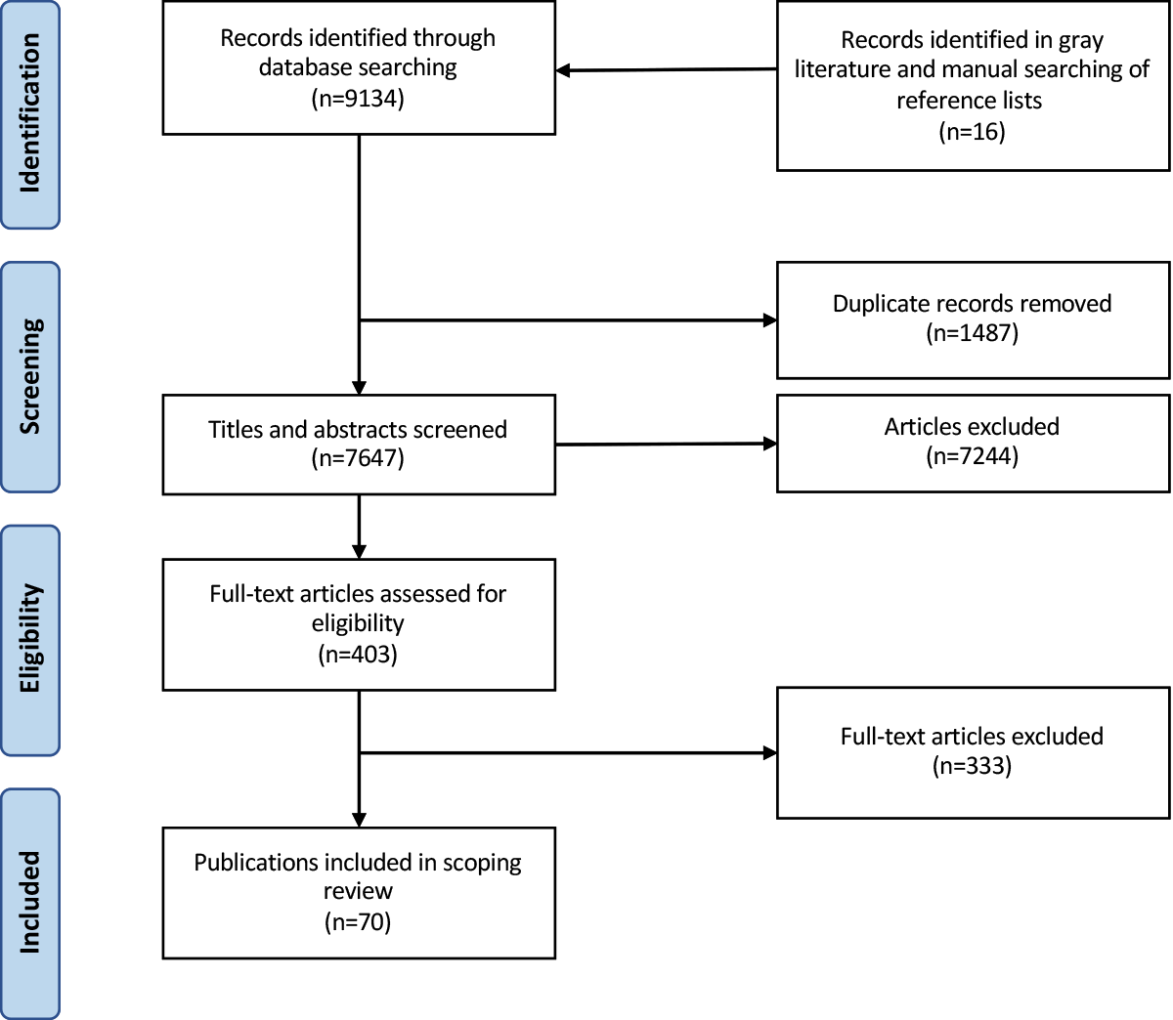
PRISMA (Preferred Reporting Items for Systematic Reviews and Meta-analyses) flow diagram.

We identified 9134 publications in our initial search. From these, 1487 duplicates were removed, leaving 7647 records for consideration. Next, titles and abstracts were screened based on our eligibility criteria, and 7244 publications were excluded. A further 333 publications were excluded during the full-text screening stage. The PRISMA flow diagram is presented in Figure 1. The full list of the included studies is provided in Multimedia Appendix 2 [16,26-95].

### Characteristics of Sources of Evidence

Of the 70 articles included in this review, 26% (18) reported positive outcomes following client participation in text-based e-mental health services, whereas only 4% (3) reported that text-based services were not effective in meeting the needs of clients. A total of 16% (11/70) of studies offered an insight into why some individuals are more or less likely to engage in text-based communications. Furthermore, 33% (23/70) analyzed text-based communications to identify predictors of mental health status, and 19% (13/70) specifically analyzed text-based communication during the course of therapy in an attempt to predict future mental health status. More studies were published in 2018 and 2019 than in any other years (n=10 and 11, respectively), and more than half of the studies (40/70, 58%) had been published in the last 5 years, with five published in 2020 (5/70, 7%). Forty-five studies (45/70, 64%) reported participants’ sex, and in the majority of those studies (34/45, 76%), a greater proportion of participants were female. The table in Multimedia Appendix 2 gives details of the number of participants, their age range, sex, background information/psychological status, and the location of the study for each source cited.

Text-based communication between clients and mental health professionals may involve SMS text messages, mobile phone apps, or internet chats. This review reveals that (1) few studies of these services have been conducted, (2) existing studies focus largely on children and adolescents in urban and suburban areas, (3) the quality of the studies varies considerably, and (4) fundamental questions remain unanswered. We first reviewed studies that reported a positive effect of text-based chat therapy on mental health outcomes.

### Effectiveness of Text-Based E-Mental Health Services

In total, 18 studies reported that text-based e-mental health services were effective in treating mental health conditions. A single session of text-based counseling reduced anxiety to a degree comparable to traditional face-to-face counseling [16]. Similarly, text-based counseling increased the level of hope in a sample of young people (16-25 years) at the 6-week follow-up compared with those who did not pursue online counseling [26].

Fukkink and Hermanns examined children’s well-being (8-18 years) after accessing the Dutch Kindertelefoon phone help line or online text-based chat service. In the first study [27], 902 children (mostly girls) completed a 2-item mental health survey before and after a session (339 chats and 563 phones). Text-based conversations lasted approximately 30 min, whereas telephone conversations lasted only approximately 8 min on average. Both counseling modes increased children’s sense of well-being and decreased their perceived problem burden. Interestingly, the effect was more pronounced for text-based chats than for telephone communication. A follow-up questionnaire completed by 223 children indicated that benefits persisted 1 month after the intervention, at which time the 2 modes did not differ. In the second study [28], which analyzed a sample of 95 conversations (53 chat and 42 telephone), both service modes improved children’s well-being and decreased their perceived problem burden.

An analysis of the Kids Help Line service available to Australian youths [29] revealed that telephone counseling sessions (45-60 min) were superior to text-based sessions (50-80 min), although text-based counseling (chosen by clients experiencing more severe mental health issues) was still rated as helpful.

A series of studies examining the text-based subscription service *Talkspace*—within which clients interact with licensed therapists via text-based chat using an app—also showed positive mental health outcomes. A feasibility study examined clinical outcomes, client satisfaction, and the quality of the therapeutic relationship among 57 questionnaire respondents who had been using Talkspace for 3-4 months [30]. Comparisons of pre- and posttherapy scores of psychiatric distress indicated clinically significant improvements in 46% (25/54) of the sample. Self-reports of client satisfaction predominantly rated text therapy as the same as or better than face-to-face therapy regarding convenience, access, effectiveness, and progress with problems. The quality of the therapeutic relationship (accounting for 30% of the variance in reported clinical improvements) was rated significantly lower than that of in-person treatments. Another study evaluated outcomes for 51 Talkspace clients (mean age 34 years, SD 9; 67% female) after 14-15 weeks of treatment and found clinically significant posttherapy symptom reduction for depression (31/38, 84%) and anxiety (25/39, 64%) [31]. Data from 267 users indicated that the service was predominantly accessed by females (198/267, 74.2%), aged 21-50 years (243/267, 91.0%; median 34 years), with a college education (212/267, 79.4%), living in (sub)urban locations (232/267, 86.9%). Past barriers to face-to-face treatment reported by 240 participants included cost (130/240, 54.2%), excessive time and inconvenience (101/240, 42.1%), and face-to-face therapy not being helpful (67/240, 27.9%) [32]. These findings indicate that Talkspace is effective in addressing a range of mental health issues, although the results should be interpreted with caution because the authors were affiliated with the service.

Evaluating 318 users’ experiences with BetterHelp, a multimodal digital psychotherapy intervention using text, video, and/or phone communication, Marcelle et al [33] found that depression symptom severity was reduced after intervention, with no differences found for gender, socioeconomic status, or self-reported physical health status. The intervention involved a significant text-based chat component as well as other modes of service delivery.

One of the most rigorous studies concerning the effectiveness of text-based counseling [34] randomly assigned 297 adults with depression (18-75 years) to online cognitive behavioral therapy (up to ten 55-min sessions) in addition to their regular medical care or to a control condition. At the 4-month follow-up (n=210), 38.1% (43/113) patients in the intervention group had recovered from depression, compared with 24% (23/97) in the control group, with therapeutic gains maintained after 8 months, providing strong evidence that e-mental health services (including text-based counseling) offer effective and lasting treatment for serious mental health conditions.

Young people with depressive symptoms (n=263) were randomly assigned to either an intervention condition (up to 5 real-time chat sessions with a trained health care professional) or a waiting list [35]. Text-based chat was more effective than waiting list control in reducing depressive symptoms, with effects increasing from follow-up to posttest, suggesting benefits extended beyond the intervention. An online cognitive behavioral therapy intervention (8 modules and 8 chat sessions over 8 weeks) was more effective than an attention counseling control condition in reducing depressive symptoms in a sample of 70 adolescents (15-19 years) [36], with improvement maintained after 6 months.

Goldin et al [37] evaluated the effectiveness of the Ascend text-based program for treating depression (8 modules over 8 weeks). Clients were primarily female (22/22, 100% in study 1; 76/95, 80% in study 2), young adults (mean age 23.2 years, SD 1.1 in study 1; mean age 32.0 years, SD 9.9 in study 2), and college educated. On average, the therapist spent 20 min chatting with each patient per week. The intervention reduced symptoms of depression, suggesting that text-based chat may be effective in augmenting therapy.

Recent evaluation of Australian crisis support service, Lifeline Text, linked text message counseling to improved mental health [38]. Help seekers with suicidal ideation and poor mental health symptoms, including anxiety, depression, or issues related to domestic and family violence, showed overall reductions in distress, increased social connection, and greater confidence in their ability to cope after the text conversation.

These studies provide compelling evidence that text-based counseling alleviates mental health issues and is effective in treating psychological distress and depression. It may also be effective for treating substance abuse [39,40], reducing high-risk behaviors [41], and improving the subjective experiences of individuals with attention deficit hyperactivity disorder and/or autism [42].

### Limitations of Text-Based E-Mental Services

Three studies did not report positive therapeutic outcomes following text-based counseling. Almost half of a sample of African American women reported that text-based interventions for depression and anxiety were not a desirable form of treatment [43]. A randomized controlled trial [44] showed that depression symptoms were reduced for a standard treatment with automated internet-delivered depression management more than with standard care plus optional text-based chat with a therapist. Despite encouragement, few users chose the chat option, making it unclear whether the chat component was ineffective or whether lack of engagement limited its usefulness. Lack of engagement was problematic in a trial involving a chat-based intervention aimed at adolescents, which was abandoned because of insufficient recruitment [45]. Interestingly, two-thirds of those who declined to participate also refused usual care; the remaining third cited preferences for face-to-face counseling.

Two previous systematic reviews [46,47], evaluating evidence from a total of 14 studies (a subset of those reviewed here), reported emerging support for the use of online text-based chat as an alternative to face-to-face and phone-delivered therapy, despite some mixed findings that can be accounted for by variability in intervention design (eg, target of treatment, length, and type of intervention). Andersson et al [48-50] argue that therapist-guided internet-delivered treatments are effective in treating a range of mental health conditions, can be as effective as face-to-face treatments, and lead to sustainable improvements. However, there will always be clients who prefer face-to-face treatment. To understand why, we next examined client experiences of text-based e-mental health services.

### Client Experiences of Text-Based Counseling

Eleven studies examined client experiences of text-based counseling and how fit and preference affect outcomes. All suggest that people generally disclose emotions similarly in computer-mediated and face-to-face communications, and there is evidence that computer-mediated communication can encourage the expression of emotion [51] and true-self qualities [52]. To understand why text-based modes of delivery might better suit some clients than others, we review studies that explored why some people are more likely to engage in text-based counseling.

For children accessing the Kindertelefoon e-mental health service [28], chat was viewed positively because children appreciated the distance (anonymity, security, privacy, and control over self-presentation), especially when discussing emotional problems. Those who preferred phone conversations perceived chat as impersonal or distant. These opposing assessments of text-based communication (private vs impersonal) are a recurring theme in studies investigating why clients do or do not engage with text-based counseling.

In an interesting case study [53], a client with severe obsessive-compulsive disorder received 40 psychotherapy sessions via videoconferencing during which the client disclosed a number of low-shame issues. Adding text chat in later sessions promoted the disclosure of clinically important information, particularly regarding feelings of shame, guilt, and embarrassment, which the client had difficulty disclosing in video-only sessions. Switching to text chat eliminated anxiety-provoking eye contact when disclosing more personally confronting topics.

Interviews with 24 patients with depression [54] were used to evaluate an online e-mental health treatment [34]. Some patients experienced difficulty expressing themselves in writing, but for others, it facilitated re-examination of thoughts, feelings, and behaviors, and reflection on therapeutic exchanges. Patients who were not comfortable with online communication disliked the less fluid interactions, reduced content covered during sessions, and waiting for therapist responses. For others, the delays created space to think, reflect, and communicate without interruption. Three studies observed that text-based chat sessions take longer than phone counseling sessions [27,29] and generate fewer words than verbal exchanges [55]. Overall, the findings suggest that when patients with depression are comfortable with online communication and with expressing their feelings in text, the anonymity of an online therapeutic relationship may be attractive, and online therapy is an acceptable and helpful alternative to face-to-face services.

### Analyzing Text-Based Communications to Identify Predictors of Mental Health Status

Studies investigating linguistic predictors of mental health status are scarce. Few have linguistically analyzed texts from individuals at risk of mental health problems. In total, 13 studies analyzed text-based therapy transcripts with the aim of predicting future mental health status. We also review 9 studies (from the much larger literature on natural language processing) that mined social media or internet forum posts with the specific aim of identifying linguistic patterns that predict mental health status because these are useful in situating the emerging text-based counseling research within the literature and offer insight into likely future directions. Recent advances in computational linguistics have increased the sophistication and complexity of analyses, enabled by the development of computational linguistic tools such as Linguistic Inquiry and Word Count (LIWC) [56], which produces statistical distributions of words within predefined and psychologically meaningful categories (eg, function words, pronouns, or verbs).

With LIWC (and similar algorithms), researchers can explore language patterns across the mental health conditions of interest. Ten studies have used LIWC to show that people with depression tend to focus on themselves. This was realized linguistically as an increased use of first-person singular pronouns (eg, I, me, my, mine) [57-59]. However, first-person singular pronouns may be a linguistic marker of general proneness to distress or negative emotionality rather than depression [60,61]. Early studies analyzed formal written texts for such patterns. Stirman and Pennebaker [62] compared 300 poems written by 18 poets, half of whom had committed suicide. Suicidal poets made more frequent use of first-person singular pronouns and less use of collective pronouns (eg, we, our). Rude et al [63] analyzed narratives written by college students who were depressed, formerly depressed, or had never been depressed. Depressed students used more first-person singular pronouns, more negative valence words, and fewer positive emotion words than those who had never been depressed. First-person singular pronoun use has been linked to the severity of depression and anxiety symptoms [64,65]. More recently, LIWC was used to examine whether first-person singular pronouns in patient interviews predict future mental health in a clinical sample of 29 inpatients undergoing psychodynamic treatment for depression [66]. First-person singular pronoun use did not predict depressive symptoms at baseline but predicted depressive symptom severity at follow-up 8 months later, especially the use of objective (*me*) and possessive (*my*) pronouns.

Eleven studies have investigated linguistic indicators of depression other than frequency of pronouns, in text data from social media platforms such as Twitter and Facebook [67] or online forums such as Reddit [68]. In social media posts, the onset of major depressive disorder can be predicted by reduced user engagement, use of negative valence words, and first-person pronouns [69]. Content words that could reliably predict depression included symptoms (eg, anxiety, withdrawal), disclosure (eg, enjoy, care), relationships (eg, home, friends), religion (eg, Jesus, bible), and treatment (eg, side effects, therapy). Regression models based on Facebook language use have been used to predict the degree [70] and incidence of depression [71].

Aspect-based sentiment analysis of online forum posts has been used to determine how individuals with different mental health conditions express themselves about various topics [72]. Al-Mosaiwi and Johnstone [73] found that internet forum users with depression, anxiety, and suicidal ideation used more absolutist words (ie, words indicating certainty). A content analysis comparing letters posted in online self-help groups for suicide, depression, and anxiety with letters posted in a control group [74] found that use of absolutist words was a better predictor of suicidal ideation than use of negative valence words or first-person pronouns [73,74] and can thus be a specific linguistic marker of depression, anxiety, and especially suicidal ideation.

Emotion words may also be predictive of mental health status. Owen et al [75] evaluated the efficacy of an online self-guided coping skills training and support intervention for women with early stage breast cancer. Greater use of words expressing anxiety, sadness (but not anger), and cognitive processing were associated with improved emotional well-being at follow-up, whereas greater expression of sadness (but not anxiety or anger) was associated with improved quality of life. This demonstrates that in a treatment context, limiting the analysis to emotion word use at a categorical level can potentially overlook subtle but meaningful differences in emotional expression.

Using more sophisticated measures that captured subtle changes in emotional state from negative emotion word instability, Seabrook et al [76] reliably predicted depression severity. They also created an emoji and internet slang supplement to the LIWC dictionary, which increased the accuracy of depression identification.

Low complexity in clients’ language has been shown to predict depression. Individuals undergoing treatment who were likely to remain depressed used fewer complex syntactic constructions such as adverbial clauses, perhaps because these require greater cognitive effort. Morales [77] also found that a number of LIWC content categories (eg, job, sad, sleep) were positively correlated with depression levels.

A recent study considered sex differences in fictional letters. Depressive symptoms were predicted by the ratios of pronouns to nouns and of verbs to nouns for men but by the ratios of finite verbs to number of sentences and of punctuation to number of sentences for women [78]. Thus, sex differences may need to be considered when identifying linguistic predictors.

In summary, there is a range of linguistic predictors at both the lexical and syntactic levels, which have been shown to correlate with mental health status and can be reliably identified with modern computational linguistic tools.

### Analysis of Text-Based Communication During the Course of Therapy

Ten studies that examined transcripts of therapeutic conversation transcripts revealed changing narrative processes during the course of treatment, and these were linked to treatment outcomes (eg, [79-81]). For example, clients’ increasing use of reflexive language and decreasing use of external language in therapeutic conversation were associated with better therapeutic outcomes [79]. Exploratory text-based analysis of therapist emails can also provide insight into therapist behaviors that support therapy adherence [82].

One study examined word use in short essays written by outpatients treated for personality disorders 3 times over 2 years of treatment [83]. The use of first-person singular pronouns, negative emotion words, causation words (eg, because, effect), past tense verbs, and future tense verbs declined over treatment, whereas the use of positive emotion and present tense words increased. However, only a reduction in the use of negative emotion words was a reliable predictor of symptom improvement.

Van der Zanden et al [84] examined 7 word categories (first-person pronouns, positive emotions, negative emotions, causation, insight, discrepancy, and social processes) in text-based chat session transcripts of participants who completed 5-6 sessions in an online group course for young adults with depressive symptoms. Only increasing the use of discrepancy words (eg, should, wish) reliably predicted depression improvement.

Dirkse et al [85] analyzed texts from adults with generalized anxiety symptoms participating in a 12-module course of therapist-assisted, internet-delivered therapy. Greater use of negative emotion, anxiety, and sadness words positively correlated with heightened anxiety ratings; greater use of negative emotion, sadness, and anger words positively correlated with heightened depression ratings; and greater use of negative emotion and anger words positively correlated with heightened panic ratings. Patients’ self-rated mental health status correlated with decreasing use of emotion, anxiety, and causation words, and increasing use of past tense words.

Using data from the York I depression study [86], Huston et al [87] examined clients’ language use in face-to-face psychotherapy sessions. They analyzed 6 word categories (positive emotion, negative emotion, causation, past focus, negation, and first-person singular pronouns) in 24 sessions, early (T1) and late (T2) for 12 clients identified as having good (n=6) and poor (n=6) treatment outcomes. No significant change in language use patterns occurred from T1 to T2, but greater use of positive emotion words at T1 was associated with good treatment outcomes and greater use of past focus words at T1 with poor treatment outcomes. Acknowledging the limitations of word count analysis, the authors highlighted the need for research using more frequent sampling to explore language patterns as potential measures of therapeutic progress.

Two studies examined large databases of client-therapist interactions [88,89]. To determine whether discourse features of text message counseling conversations could predict clinical outcomes, Althoff et al [88] examined a large data set (approximately 80,000 conversations of approximately 40 messages each). After the session ended, clients’ answers to *How are you feeling now?* became their ground-truth labels. Clients with a smaller amount of self-focus tended to have more successful conversations. Tay [89] examined transcripts from 4 client-therapist dyads each lasting 20 sessions (472,009 words) that were taken from the counseling and psychotherapy transcripts, client narratives, and reference works.

A study of an internet-delivered pain management intervention for adolescents with chronic pain [90] used text mining and analytic techniques to messages between coaches and patients and successfully identified messages that raised concerns. Cluster analysis identified subgroups of individuals with common communication and engagement patterns that could be used to tailor interventions. Such techniques could conceivably also be applied to e-mental health services.

Calvo et al [91] offered a comprehensive review of natural language processing in mental health applications. Their discussion of computational linguistic analyses reveals that demographic, linguistic, behavioral, and social data may be combined to construct sophisticated models to identify at-risk individuals. Although no study to date has succeeded in the ultimate goal of accurately predicting suicide risk and guiding mental health professionals to intervene to save lives, progress has been made in recent years. A study by Ruiz et al [92] built 6 machine learning models to predict suicide risk levels from posts on the Reddit forum using a large training data set of 31,553 posts from 496 users. Naive Bayes was the most accurate model for flagging urgent suicide risks.

Quantitative measures of therapist emails can also shed light on the key aspects of program delivery and client adherence to eHealth programs [82]. Content analysis of 490 therapist emails to clients showed that therapist behaviors were represented by 8 behavior codes: deadline flexibility, task reinforcement, alliance bolstering, task prompting, psychoeducation, self-disclosure, self-efficacy shaping, and empathetic utterances. Client adherence to the program was supported by therapist communication that supported deadline flexibility and task reinforcement.

In summary, modern computational linguistic tools and techniques allow analyses that may help predict progress during treatment and may ultimately identify individuals at risk. They also offer an opportunity to quantitatively assess the interrelationship between therapist behaviors, program adherence, and success.

## Discussion

### Principal Findings

The aim of this scoping review was to map the research that has explored text-based e-mental health counseling services and studies that have used language use patterns to predict mental health status. One of the aims of this study was to assess whether text-based counseling with a therapist could offer an e-mental health solution appropriate for underserved populations, such as rural and remote communities. Of the 9334 reviewed records, 70 met the eligibility criteria.

Although more research is needed to understand the nuances and complex interactions necessary for personalized care, converging evidence shows that text-based counseling services and interventions are effective in treating a variety of mental health conditions. This review of the relevant literature gives reason to be optimistic regarding the potential for text-based interventions to contribute to addressing the mental health needs of underserved populations. Evidence suggests that text-based counseling produces desirable outcomes for a range of mental health conditions for clients willing to engage with this delivery mode.

However, there is a dearth of research concerning its applicability to underserved communities, including those living in rural and remote regions. Our review has revealed that the characteristics of text-based approaches to service delivery (longer sessions, fewer words transmitted, more anonymous or impersonal interactions) are perceived positively by some cohorts but negatively by others, and this perception is likely to affect engagement with such services. This led Hoermann et al [47] to suggest that *if this mode of intervention delivery generates similar effectiveness but longer session times, generating fewer words, and in some groups, lower satisfaction, this draws into question the clinical practicality of this mode of delivery.* However, our focus on identifying a desirable delivery mode of mental health services for people living in rural and remote communities leads us to a different conclusion. First, if text-based communications are effective, they can provide much needed mental health services. Second, for certain segments of the population, the supposed limitations of text-based communication (slower communication, facelessness, and lack of voice) may actually be advantageous or desirable. Increased anonymity and privacy may make some individuals more likely to engage and reveal more truthful information. Some therapists also prefer chat sessions because they allow editing outside the client’s view; thus, minimizing possible awkwardness when fumbling for words in face-to-face communication [16]. Thus, text-based counseling has the potential to both increase access to mental health services and improve clinician-client relationships.

It is important to acknowledge that text-based counseling requires a level of technology literacy, and this may present a barrier for some clients to engage with such e-mental health services (and this is likely to correlate with age). Service providers should heed this limitation by designing their services in accessible ways that minimize the burden for clients who lack technical expertise (eg, SMS may be more accessible than internet chat). Relatedly, high-cost services will not reach (and therefore not help) some of the population groups that are both high need and high risk. Many such groups have lower socioeconomic status and incomes and are unlikely to be able to afford or engage in high-cost or subscription-based approaches to treatment.

Finally, the transcripts of text-based therapy-related communications not only permit reflection on the part of the client but also allow researchers to conduct linguistic analyses to predict future mental status. The potential applications of an accurate, scalable approach to mental health are far-reaching, with implications for early screening and targeted interventions.

### Future Directions in Analyses of Text Data

Computational linguistic techniques will continue to evolve as more sophisticated tools are developed to overcome the current limitations. For example, LIWC is unable to detect or compute context and cannot account for changes in meaning resulting from irony, sarcasm, or idioms [93], nor does it take into account negations [87] or qualifiers before words. Predefined LIWC dictionaries may not be sufficiently broad to account for the categories of interest [93]. Although LIWC does not permit the creation of new categories, new dictionaries can be added. Thus, some limitations can be overcome by using dictionaries composed of nonambiguous adjectives and expanded by a distributional strategy [94], whereas others may be addressed by supplementing LIWC with computational linguistic techniques such as unsupervised natural language processing models. To date, researchers must work around these limitations to conduct computational linguistic analyses of text data.

As chat transcripts automatically document the therapeutic process, the text may be used to predict future mental health status and identify at-risk individuals. The accuracy of such predictive models depends on the integrity of the training data. A biased data set affects the accuracy of the predictions or classifications the model makes. Another concern is how false negatives are dealt with, particularly in cases where an individual’s safety is at risk. This is an important consideration to bear in mind when such techniques are used to augment the diagnosis or treatment. It is also worth noting that the ethical and privacy risks associated with text-based counseling can be mitigated by using encryption and deidentifying transcripts (eg, names of people and places).

In addition, there is evidence that linguistic patterns in children differ from those in adults [95]. Therefore, it should not be assumed that predictive relationships between linguistic patterns and mental health status will apply equally across the lifespan. More research is needed to understand how developmental differences influence these predictive relationships.

### Limitations of the Study

This scoping review covers a growing area of research, and thus fundamental questions need to be answered to advance the field. Are findings in one population or region transferable to another (eg, from adolescents in urban areas to adults in rural areas)? What obstacles might potentially limit such generalizability? In addition, the scope here is limited to the literature documenting text-based e-mental health services and is not an evaluation of the technologies used by those services. Given the increased development of mobile apps for many services, a review of e-mental health apps following the MARS protocol [96] would complement this work.

### Conclusions

The review reveals a clear need for intensified research into how evidence-based e-mental health practices can reach and serve clients. This is especially important for communities where mental health services are limited, and individuals must overcome multiple barriers to help seeking (as is the case in rural and remote areas). This need is likely to increase as governments and organizations respond to the COVID-19 pandemic by exploring flexible working arrangements that include remote working and exacerbate social isolation due to social distancing policies.

Further investigation is required to determine whether transcripts of text-based communication between clients and therapists can be used to accurately predict mental health outcomes. Our review of the computational linguistic literature on text-based predictors of mental health suggests that large-scale automatic screening of mental illness and identification of at-risk individuals may soon be possible.

## Appendix

Multimedia Appendix 1 PRISMA-ScR (Preferred Reporting Items for Systematic reviews and Meta-analyses extension for Scoping Reviews) checklist.

Multimedia Appendix 2 Study information.

## Abbreviations

LIWC: Linguistic Inquiry and Word Count
PRISMA: Preferred Reporting Items for Systematic Reviews and Meta-analyses
