# Supplementary material for: Suitability of Text-Based Communications for the Delivery of Psychological Therapeutic Services to Rural and Remote Communities: Scoping Review
Source: JMIR Ment Health. 2021 Feb 24;8(2):e19478. doi: 10.2196/19478 (PMC7946577; doi:10.2196/19478)
Supplement: Multimedia Appendix 2 [file mental_v8i2e19478_app2.docx]

**Multimedia Appendix 2**. Study information.

| Study | Country | Location | *n* (condition) | *M* Age (range) | Gender | Background/Psychological status |
| --- | --- | --- | --- | --- | --- | --- |
| Cohen 1999 [16] | USA | Urban | 24 (clients) | undergraduates |  | Anxiety |
|  |  |  | 6 (counsellors) | graduate students | 100% m |  |
| Dowling 2015 [26] | Australia | Urban | 152 | 17.0 (16-25) | 87.8% f | Clients of an online mental health service. |
| Fukkink 2009 [27] | Netherlands | Urban | 339 (chat) | 13.8 (8-18) | 80% f | Clients of Kindertelefoon service |
|  |  |  | 563 (phone) | 12.0 | 71% f |  |
| Fukkink 2009 [28] | Netherlands | Urban | 53 (chat) | (9-17) | 84% f | Clients of Kindertelefoon service |
|  |  |  | 42 (phone) |  | 77% f |  |
| King 2006 [29] | Australia |  | 86 (online) | 15.4 | 95% f | Clients of Kids Help Line service |
|  |  |  | 100 (phone) | 13.1 | 66% f |  |
| Hull 2017 [30] | USA |  | 57 | 34.1 (18-60) | 67% f | Clients of an SMS text therapy service |
| DellaCrosse 2019 [31] | USA | 86.9% (sub)urban | 51 | 34 (18-55) | 67.3% f | Talkspace clients reporting depression/anxiety |
| Nitzburg 2019 [32] | USA |  | 267 | 34.3 (18-68) | 74.2% f | Depression or anxiety symptoms |
| Marcelle 2019 [33] | USA |  | 318 | 33.3 (19-72) | 79.9% f | BetterHelp clients reporting sadness, grief, or depression |
| Kessler 2009 [34] | UK |  | 297 | 18-75 | 69% f | Depression RCT |
| Kramer 2014 [35] | Netherlands |  | 131(chat) | 19.4 (12-22) | 79.4% f | Depression (CES-D ≥ 22) RCT |
|  |  |  | 132 (wait list) | 19.6 | 78.0% f |  |
| Topooco 2018 [36] | Sweden |  | 33 (iCBT) | 17.2 (15-19) | 93.9% f | Depression (BDI-II ≥ 14) RCT |
|  |  |  | 37 (control) | 16.9 | 94.6% f |  |
| Goldin 2019 [37] | Finland |  | 22 (Study 1) | 23.2 | 100% f | Clients of Ascend with depression (BDI-II > 9) |
|  |  |  | 95 (Study 2) | 32.0 | 80.0% f | Clients of Ascend with depression (PHQ-9 > 4) |
| Williams 2020 [38] | Australia | 89.8% (sub)urban | 3,236 | 26.1 (11-71) | 79.6% f | Clients of Lifeline Text |
| Blankers 2011 [39] | Netherlands |  | 205 | 42.0 | 50% f | Problem alcohol use (>140g alcohol/week) RCT |
| Schaub 2015 [40] | Switzerland |  | 114 (chat) | 28.4 | 69.3% m | Problem cannabis use RCT |
|  |  |  | 101 (self-help) | 30.2 | 76.2% m |  |
|  |  |  | 93 (wait list) | 31.0 | 82.0% m |  |
| Lelutiu-Weinberger 2015 [41] | USA |  | 41 | 25.2 (18-29) | 100% m | HIV risk in young men |
| Wentz 2012 [42] | Sweden |  | 12 | 19.6 (15-26) | 58.3% m | ADHD and ASD |
| McCall 2020 [43] | USA | Urban | 101 | 38.9 | 100% f | Anxiety and depression |
| Kordy 2016 [44] | Germany |  | 77 (SUMMIT) | 18-65 |  | Depression RCT |
|  |  |  | 79 (SUMMIT-P) |  |  |  |
|  |  |  | 80 (control) |  |  |  |
| Crutzen 2014 [45] | Netherlands |  | 12 | adolescents |  | Psychosocial problems RCT |
| Dowling 2013 [46] |  |  | 6 studies |  |  | Systematic review of interventions using text-based chat |
| Hoermann 2017 [47] |  |  | 24 studies |  |  | Systematic review of interventions using text-based chat |
| Andersson 2016 [48] |  |  |  |  |  | Review of internet-delivered psychological treatments |
| Andersson 2019 [49] |  |  |  |  |  | Review of 9 meta-analyses of internet interventions |
| Andersson 2018 [50] |  |  |  |  |  | Review of iCBT studies |
| Derks 2008 [51] |  |  |  |  |  | Review of online emotion communication |
| Bargh 2002 [52] | USA |  | 46 (Exp1) | undergraduates | 60.9% f | Showing “true self” on the internet |
|  |  |  | 36 (Exp 2) |  | 50.0% f |  |
|  |  |  | 20 (Exp 3) |  | 50.0% f |  |
| Stubbings 2015 [53] | Australia |  | 1 | 22 | 100% m | OCD case study |
| Beattie 2009 [54] | England | Urban + semi-rural | 24 | (24-66) | 70.8% f | Depression |
| Rodda 2014 [55] | Australia |  | 1722 (chat) | 34.5 | 60.6% m | Problem gamblers |
|  |  |  | 299 (email) |  | 53.8% m |  |
| Tausczik 2009 [56] |  |  |  |  |  | LIWC methods |
| Fast 2010 [57] | USA |  | 181 | undergraduates | 50.3% m | Depressive symptoms |
| Pyszczynski 1987 [58] |  |  |  |  |  | Theoretical paper |
| Weintraub 1981 [59] |  |  |  |  |  | Book |
| Lyons 2018 [60] | USA |  | 600 posts |  |  | Predicting mental distress categories from Reddit posts |
| Tackman 2019 [61] | USA |  | 253 | 19.1 (19-43) | 60.1% f | Depression and negative emotionality |
|  | USA |  | 133 | 40.6 | 63.6% f |  |
|  | USA |  | 133 | 43.1 | 70.7% f |  |
|  | USA |  | 161 | 19.5 | 73.1% f |  |
|  | Germany |  | 305 | 28.6 | 57% f |  |
|  | Germany |  | 55 | 46.0 | 69.1% f |  |
|  | USA |  | 110 | 20.0 | 57.3% f |  |
|  | USA |  | 937 |  | 61% f |  |
|  | USA |  | 948 | 19.0 | 61.3% f |  |
|  | USA |  | 1,269 |  | 65.8% f |  |
| Stirman 2001 [62] |  |  | 9 (suicidal poets) | 41.3 (30-58) | 55.6% m | Predicting suicide from word use |
|  |  |  | 9 (nonsuicidal poets) | 43.3 (32-59) | 55.6% m |  |
| Rude 2004 [63] | USA |  | 31 (depressed) | 18.0 | 93.5% f | Word use in depression |
|  |  |  | 26 (prev depressed) | 19.0 | 76.9% f |  |
|  |  |  | 67 (never depressed) | 18.8 | 70.1% f |  |
| Molendijk 2010 [64] | Netherlands |  | 110 (depressed) | 38.6 | 55.0% f | Word use in personality disorder and depression |
|  |  |  | 90 (prev depressed) | 37.9 | 54.0% f |  |
|  |  |  | 104 (never depressed) | 37.3 | 57.0% f |  |
|  |  |  | 108 (controls) | 37.6 | 57.0% f |  |
| Brockmeyer 2015 [65] | Germany |  | 25 (anorexia) | 24.6 (18-45) | 100% f | Word use in Anorexia nervosa |
|  |  |  | 29 (chronic depressed) | 39.2 (18-60) | 62% f | Word use in depression |
|  |  |  | 30 (nonchronic) | 38.6 | 67% f |  |
|  |  |  | 29 (controls) | 38.3 | 60% f |  |
| Zimmermann 2017 [66] | German |  | 29 | 38.5 (19-59) | 70.0% f | Word use in depression |
| Guntuku 2017 [67] |  |  |  |  |  | Review of predicting depression via social media |
| Park 2018 [68] |  |  | 41,967 Reddit users |  |  | Predicting depression, anxiety, PTSD from Reddit posts |
| De Choudhury 2013 [69] |  |  | 476 Twitter users |  | 51.1% m | Predicting depression from Twitter posts |
| Schwartz 2014 [70] |  |  | 28,749 Facebook users | 24.8 | 57.0% f | Predicting depression from Facebook posts |
| Babu 2018 [71] |  |  | 1,000 Facebook users |  |  | Predicting depression from Facebook posts (Thesis) |
| Davcheva 2018 [72] |  |  | 1,155,403 posts |  |  | Predicting mental disorders from mental health forum posts |
| Al-Mosaiwi 2018 [73] |  |  | 6,400 forum users |  |  | Word use in depression, anxiety, and suicidal ideation |
| Fekete 2002 [74] | USA + other |  | 78 (suicide ideation) | (18-40) | 69.9% m | Depression, anxiety, and suicidal ideation |
|  |  |  | 51 (depression) |  |  |  |
|  |  |  | 50 (panic) |  |  |  |
|  |  |  | 43 (control) |  |  |  |
| Owen 2005 [75] |  |  | 32 (treatment) | 52.5 | 100% f | Coping with breast cancer |
|  |  |  | 30 (control) | 51.3 | 100% f |  |
| Seabrook 2018 [76] | Australia |  | 29 Facebook users | 32.8 (19-45) | 60.7% f | Predicting depression from Facebook and Twitter posts |
|  |  |  | 49 Twitter users | 35.0 (16-57) | 66.7% f |  |
| Morales 2018 [77] |  |  | 84 | 31.5 (18-63) |  | Word use and depression |
| Havigerová 2019 [78] | Czech Republic |  | 172 |  | 51.7% f | Word use and depression |
| Banham 2015 [79] | Australia |  | 42 (therapists) | 28.7 (22-53) | 90.5% f | Depression |
|  |  |  | 349 (clients) | 31.6 (18-64) | 70.5% f |  |
| Bento 2014 [80] | Canada |  | 1 (good outcome) | 27 | 100% f | Depression case study |
|  |  |  | 1 (poor outcome) | 43 | 100% m |  |
| Reyes 2008 [81] | Chile |  | 1 (psychodynamic tx) | 29 | 100% f | Separation anxiety |
|  |  |  | 1 (psychodynamic tx) | 38 | 100% f | Grief |
|  |  |  | 1 (social construct) | 38 | 100% f | Interpersonal relationships |
| Arntz 2012 [83] | Netherlands |  | 299 (disordered) | 38.1 (18-65) | 56.5% f | Word use and personality disorders |
|  |  |  | 108 (controls) |  | 57.4% f |  |
| Van der Zanden 2014 [84] | Netherlands |  | 234 | 20.0 | 85.0% f | Word use and depression |
| Dirkse 2015 [85] | Canada |  | 59 | 41 (21-67) | 62.7% f | Word use and anxiety |
| Greenberg 1998 [86] | UK |  | 34 | 39.64 | 73.5% f | Depression |
| Huston 2019 [87] | UK |  | 6 (good outcome) |  |  | Word use and depression |
|  |  |  | 6 (poor outcome) |  |  |  |
| Althoff 2016 [88] | USA |  | 15,555 conversations |  |  | Word use and mental health |
| Tay 2020 [89] | USA |  | 472,009 words |  |  | Word use and mental health |
| Chen 2019 [90] | USA |  | 123 | 14.7 (11-17) | 78.9% f | Word use and chronic pain |
| Calvo 2017 [91] |  |  |  |  |  | Review of predicting mental health from non-clinical texts |
| Ruiz 2019 [92] |  |  | 9,610 posts |  |  | Predicting suicide risk from Reddit posts |
| Calvo 2010 [93] |  |  |  |  |  | Review of affect detection |
| Losada 2020 [94] |  |  | 531,582 posts |  |  | Predicting depression from Reddit posts |
| Jones 2020 [95] | UK |  | 16 | (11-17) | 75% f | Word use and depression |
